# Inflammatory lesions and brain tumors: is it possible to differentiate them based on texture features in magnetic resonance imaging?

**DOI:** 10.1590/1678-9199-JVATITD-2020-0011

**Published:** 2020-09-04

**Authors:** Allan Felipe Fattori Alves, José Ricardo de Arruda Miranda, Fabiano Reis, Sergio Augusto Santana de Souza, Luciana Luchesi Rodrigues Alves, Laisson de Moura Feitoza, José Thiago de Souza de Castro, Diana Rodrigues de Pina

**Affiliations:** 1 Department of Physics and Biophysics, Botucatu Biosciences Institute, São Paulo State University (UNESP), Botucatu, SP, Brazil.; 2 Department of Radiology, School of Medical Sciences, University of Campinas (Unicamp), Campinas, SP, Brazil.; 3 Department of Tropical Disease and Imaging Diagnosis, Botucatu Medical School, São Paulo State University (UNESP), Botucatu, SP, Brazil.

**Keywords:** Medical imaging, Image processing, Inflammation, Tumor, Magnetic resonance imaging

## Abstract

**Background:**

Neuroimaging strategies are essential to locate, to elucidate the etiology, and to the follow up of brain disease patients. Magnetic resonance imaging (MRI) provides good cerebral soft-tissue contrast detection and diagnostic sensitivity. Inflammatory lesions and tumors are common brain diseases that may present a similar pattern of a cerebral ring enhancing lesion on MRI, and non-enhancing core (which may reflect cystic components or necrosis) leading to misdiagnosis. Texture analysis (TA) and machine learning approaches are computer-aided diagnostic tools that can be used to assist radiologists in such decisions.

**Methods:**

In this study, we combined texture features with machine learning (ML) methods aiming to differentiate brain tumors from inflammatory lesions in magnetic resonance imaging. Retrospective examination of 67 patients, with a pattern of a cerebral ring enhancing lesion, 30 with inflammatory, and 37 with tumoral lesions were selected. Three different MRI sequences and textural features were extracted using gray level co-occurrence matrix and gray level run length. All diagnoses were confirmed by histopathology, laboratorial analysis or MRI.

**Results:**

The features extracted were processed for the application of ML methods that performed the classification. T1-weighted images proved to be the best sequence for classification, in which the differentiation between inflammatory and tumoral lesions presented high accuracy (0.827), area under ROC curve (0.906), precision (0.837), and recall (0.912).

**Conclusion:**

The algorithm obtained textures capable of differentiating brain tumors from inflammatory lesions, on T1-weghted images without contrast medium using the Random Forest machine learning classifier.

## Background

Inflammatory lesions and tumors are common brain diseases that present a similar pattern of a cerebral ring enhancing lesion on MRI, leading to misdiagnosis on neuroimaging [1]. They may produce severe complications, disability, and economic burden. Inflammatory lesions, particularly neuroinfections, affect millions of people worldwide, especially in low-income countries and can be caused by diverse etiological agents (bacteria, fungi, viruses, and parasites). In 2018, 296,851 new cases of brain cancer were diagnosed worldwide, which accounts for 1.6% of all cancer cases [2]. 

Depending on the type of disorder and location, inflammatory lesions and tumors present similar symptoms such as headache, dizziness, vertigo, change of humor, nausea, fainting, and coma [1]. Focal neurological signs are often found in patients with inflammatory and tumoral lesions [3]. The diagnosis of these neurological disorders considers patient history, symptoms, physical and neurological examinations. Blood analysis, cerebrospinal fluid (CSF), biopsy, and neuroimaging are fundamental. Neuroimaging modalities including magnetic resonance imaging (MRI), computed tomography (CT) and positron emission tomography (PET) provide localization, determination of etiology, and the follow up of these diseases [4, 5].

Among these, MRI presents the best soft tissue contrast detection and diagnostic sensitivity. The acquisition of MRI sequences allows the distinctive visualization of brain anatomy with different contrast of structures [6]. Perfusion sequences in MRI can be used in this differential diagnosis since they provide important physiologic and pathophysiologic parameters and can be assessed non-invasively. There are several techniques to obtain perfusion-related parameters using endogenous contrast methods or, more robustly and more widely used, exogenous gadolinium-based contrast agent dynamic methods [7]. 

Despite these qualities, MRI cannot always distinguish between tumors and inflammation due to similar imaging characteristics. In addition, many patients have contraindication to gadolinium, and inflammatory lesions, such as neurotuberculosis, may show high perfusion, leading to misdiagnosis [7]. Thus, the potential use of other tools must be investigated to differentiate inflammatory from tumoral lesions.

 For this reason, a reliable diagnosis also depends on histopathological examination of biopsy samples obtained through invasive procedures, such as surgery [8-10]. Due to these limitations, and aiming at non-invasive diagnostic aid, texture analysis extracted from medical imaging [11] has been progressively used to assist radiologists in the diagnosis of tumors [12-16] and inflammatory lesions [17-19]. 

In this study, our aim was to combine different texture analysis with machine learning to classify MRI brain lesions in two categories: inflammatory and tumoral lesions. The texture features we utilized were gray-level co-occurrence matrix (GLCM) and gray-level run length (GLRL). Some features were extracted after a pre-processing with a Wavelet’s transform. The supervised classification was achieved with machine learning (ML) approaches: support vector machine (SVM), k-nearest neighbors (kNN), and Random Forest. 

## Methods

### Image bank

The local institutional ethics committee approved this study according to our country regulations. Retrospective examinations were collected from a single 1.5 Tesla MRI scanner in a Medical School Hospital. Patients were selected according to the following criteria. 

Inclusion criteria:

- patients who underwent MRI exams between 2010 and 2020;
- patients with the diagnosis of inflammatory or tumoral lesions with a pattern of a cerebral ring enhancing lesion and non-enhancing core (which may reflect cystic component or necrosis) on MRI;
- patients with diagnosis confirmed by histopathological or CSF analysis examinations and follow-up exams.

Exclusion criteria:

- patients who had brain biopsy or surgery before the MRI acquisition;
- lesions smaller than 10 mm, MRI detecting scolex in cases of neurocysticercosis;
- patients with intracranial malformations.

The selection resulted in a database with 67 patients, being 30 cases of inflammatory and 37 tumoral lesions. Five different MRI sequences were used for feature extraction, T1- and T2-weighted spin-echo sequences, T1 with contrast medium, diffusion-weighted image sequence, and fluid attenuated inversion recovery (FLAIR). For simplicity, we will refer to the five MRI sequences with their abbreviations such as T1, T1C+, T2, DWI, and FLAIR. The complete list with all pathologies that were selected for this study, with the number of patients, and their mean lesion size is shown in Table 1. More information on patient’s ages, gender and diagnostic evaluations are presented in the Additional files 1 and 2.

**Table 1.** Complete list of all pathologies that were selected for the present study, with the number of patients (n), their mean lesion size and standard deviation (SD) in millimeters.

Brain pathology	Subtypes	Number of patients (n = 67)	Mean lesion size ± SD (mm)
Inflammatory lesions	Aspergillosis	2	31.86 ± 19.86
	Cryptococcosis	2	15.01 ± 3.81
	Neurocysticercosis	3	18.85 ± 8.70
	Neuromyelitis	1	2.90
	Pyogenic brain abscess	3	22.61 ± 9.70
	Septic-embolic brain abscess	2	14.03 ± 5.33
	Toxoplasmosis	8	24.20 ± 13.02
	Multiple sclerosis	3	12.10 ± 5.15
	Progressive multifocal leukoencephalopathy	1	7.14
	Vasculitis	1	6.87
	Tuberculous brain abscess	4	4.94 ± 2.86
	Total inflammatory lesions	30	
Brain tumors	Anaplastic astrocytoma (grade III)	11	40.95 ± 12.77
	Anaplastic ependymoma	1	12.30
	Glioblastoma (grade IV)	15	50.74 ± 11.19
	Gliosarcoma	2	25.60 ± 3.65
	Low-grade astrocytoma (grade II)	8	36.60 ±15.81
	Total brain tumors	37	

MRIs were performed using a 1.5 T Phillips Scanner with T1 and T2 acquisitions in three orthogonal planes, including T1-weighted SE gadolinium-enhanced images. MRI acquisition parameters were described as follows. Sagittal T1 spin echo, 6 mm thick, 180° flip angle; repetition time (TR) = 430 milliseconds, echo time (TE) = 12 milliseconds, matrix 200 × 350, field of view (FOV) = 25 × 25 cm. T2-weighted and proton density "fast spin echo" (FSE), 3 mm thick, 160° flip angle; TR = 4.800 milliseconds, TE = 108/18 milliseconds, matrix 256 × 256, FOV = 22 × 22 cm. Axial T1-weighted spin echo (SE): TR = 540 milliseconds, TE = 28 milliseconds. Axial T2-weighted fluid-attenuated inversion recovery (FLAIR) images TR = 8.500 milliseconds and 2.000 or 100 milliseconds, and 2.200 milliseconds, TE = 72 or 90 milliseconds, matrix of 256 × 296 and FOV of 22 × 22 cm. T1-weighted SE gadolinium-enhanced images were obtained in three orthogonal planes and T1 sagittal volumetric isotropic images. Diffusion weighted images (TR = 22ms, TE = 9ms, FOV = 230 × 250 mm), all acquired using a standard 8-channel head coil and with b value = 1000.

### Algorithm for feature extraction

Texture analysis (TA) is described as techniques that enable to quantify the variations in pixel intensity including some imperceptible to the human visual system. TA includes the quantification of the gray-level patterns, pixel interrelationships, and the spectral properties of an image. All texture analysis were processed in Matlab software. The first features extracted were the GLCM which considers the relationship between pixel pairs and registers the frequency of various gray-level combinations within a region of interest [11]. 

After we extracted the GLRL features that also evaluates the intensity relation of neighbor pixels. GLRL computes the coarseness of texture in a predetermined direction. Each set of consecutive collinear pixels in a given direction composes a gray-level run. Runs are computed in different directions in the image and relates to the number of gray-level runs for each given gray level. For both GLCM and GLRL the maximum number of gray levels considered were scaled down from 16 bits to 8 bits [11]. 

**Figure 1.**
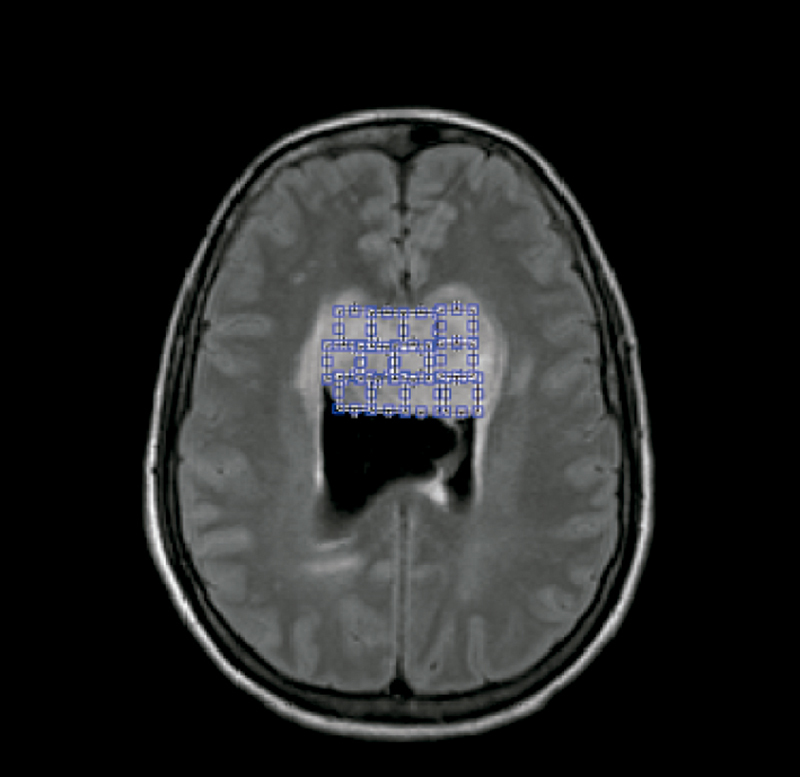
Single slice of a FLAIR weighted image of brain tumor showing the positioning of ROIs within lesions.

In the first step of this approach, we developed a computational algorithm that allows the user to open DICOM images, select slices, position ROI's and extract features. All algorithm steps were performed using Matlab software R2017a. Two radiologists with more than 15 years of experience were used as operators. They analyzed all MRI sequences, in axial orientation: T1, T1C+, T2, fluid attenuated inversion recovery (FLAIR) and DWI. Images had different sizes and were all resized to 240 x 240 pixels. The radiologists selected the slice in which the lesion appeared with its largest diameter. Regions of interest (ROI) were positioned within each lesion, including the solid and necrotic portion of the lesion and excluding the edema region, when existed. Each ROI had 10 x 10 pixels. An example of ROI positioning is presented in Figure 1. 

Statistical texture features such as mean, standard deviation, entropy, kurtosis, skewness, and correlation were extracted from those ROIs. There were 63 features including GLCM, GLRL, and Wavelet’s Transform methods. The extraction methods used are based on the relationship between pixels and gray-level frequencies within the ROI, and analyses pixels pairs, consecutive collinear pixels, and spatial frequencies at multiple scales in four different directions (0, 45, 90, and 135 degrees). For the lesions that permitted the inclusion of multiple ROI, we used the mean value of each feature. 

The features extracted were processed in the software Orange Canvas® for the application of ML classifiers. In order to determine the best approach we compared five types of image: T1, T1 with intravenous contrast (T1 C+), T2, FLAIR, and DWI. To each image, we input the data in two ways: all extracted features as raw data and only the five best-ranked features. To rank the features, we used information gain ratio and Gini Index scoring methods [20]. These feature selection methods measure the relationship between the feature and the output outcome creating a score to differentiate both groups.

We used three different methods of ML: support vector machine (SVM), k-nearest neighbors (kNN), and Random Forest. Those three methods belong to a supervised class of machine learning. In our approach, all supervised learning methods utilized texture features extracted from regions of interest to characterize the difference between inflammatory and tumoral lesions. All three methods used the textural features to train and then test data in a 10-fold cross-validation procedure. The training set was composed of 75% of all the input data and the test with 25% of input data [21]. The parameter used for each ML method was: for kNN, we selected 5 neighbors, with metric Euclidean and weight uniform; for SVM we selected the Kernel RBF with cost 1 and regression loss epsilon 0.10, and for Random Forest we selected 10 trees and did not split subsets smaller than 5.

We used statistical quantities to determine how efficiently the model classified our groups. Those quantities describe the diagnostic performance of the classification. Each classification follows a binary rule that allows four possible outcomes: true positive, true negative, false positive and false negative. From those quantities, we determined the following parameters: area under the receiver operating characteristic (ROC), curves (AUC), accuracy (CA), F-score (F1), precision and recall [22]. 

## Results

The mean age and standard deviation of our patients were 39 ±14 years in the inflammatory group and 47 ± 17 years in the tumoral group. The 63 features extracted from each patient were divided in four different categories and presented in Table 2. To increase classification scores, all 63 extracted features were analyzed with the statistical methods information gain ratio and Gini Index to select the five best-ranked features. Those two index selected which features distinguish with higher precision, recall and AUC the data between our two patient groups. Those five best-ranked were different in each MRI sequence, however wavelet entropy (EntropyWv) and the mean intensity of pixels remained constant in all five groups. All five-best ranked features are present in Table 3.

**Figure 2.**
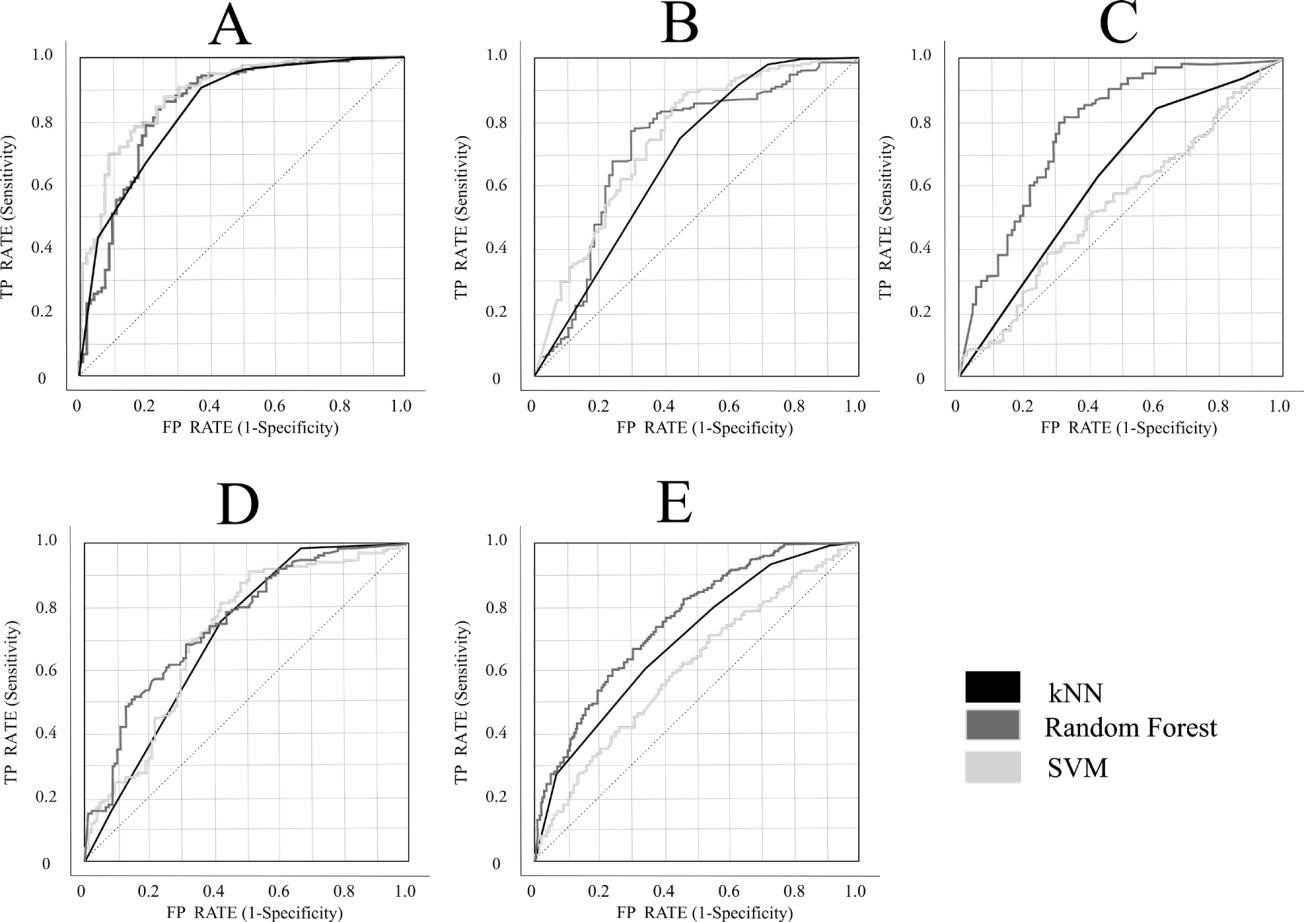
ROC curves of kNN, SVM, and Random Forest analysis. The classifiers on image **(A)** are applied to all images. **(A)** ROC curve from a T1-weighted image with the five best-ranked features. **(B)** ROC curve from T1 C+ image with the five best-ranked features. **(C)** ROC curve from T2-weighted image with the five best-ranked features. **(D)** ROC curve from DWI with the five best-ranked features. **(E)** ROC curve from FLAIR image with the five best-ranked features.

After the classification, we determined some quantities that described how well the machine learning methods performed. Those quantities were presented in Table 4: AUC, CA, F1, precision, and recall. To each of the three ML methods, we obtained a receiver operating characteristic (ROC) curves. Figure 2 displays the ROC curves of the 5 best-ranked features within each MRI image. Random Forest had the best performance among the three classifiers. 

**Table 2.** Gray level co-occurrence matrix (GLCM), gray level run-length (GLRL) and Wavelet’s transform features.

Method	Texture feature parameters
Features	Mean, standard deviation, entropy, kurtosis, skewness and correlation
GLCM	Gray co-matrix, mean, standard deviation, entropy, kurtosis, skewness, correlation, contrast, variance, sum average, sum variance, sum entropy, difference variance, difference entropy, information measures of correlation, autocorrelation, dissimilarity, homogeneity, cluster prominence, cluster shade, maximum probability, inverse difference, inverse difference normalized, and inverse difference moment normalized.
GLRL	Short run emphasis (SRE), long runs emphasis (LRE), gray level non-uniformity (GLN), run percentage (RP), run length non-uniformity (RLN), low gray level run emphasis (LGRE) and high gray level run emphasis (HGRE)
Wavelet’s transform	wEntropy, energy ‘sym4’ (Ea, Eh, Ev, Ed, E_soma), energy ‘haar’ (Ea, Eh, Ev, Ed, E_soma), energy ‘bior’ (Ea, Eh, Ev, Ed, E_soma)

**Table 3:** The five best-ranked features in each MRI sequence, being T1-weighted sequence (T1), T1-weighted sequence with contrast medium (T1C+), T2-weighted sequence, diffusion-weighted image sequence (DWI), and FLAIR.

Five-best ranked features					
Images	Features				
*T1*	EntropyWv	Mean	Ed_haar_2	Ev_haar_2	Ev_sym4_2
*T1C+*	Mean	EntropyWv	Ed_sum4_2	Ed_haar_1	Ea_bior3.3
*T2*	Mean	EntropyWv	Ed_bior3.3_1	Ev_bior3.3_1	E_soma_haar_2
*DWI*	Ed_haar_1	Mean	Ed_bior3.3_2	EntropyWv	E_soma_bior3.3_1
*FLAIR*	Mean	EntropyWv	Ed_bior3.3_1	Ea_haar	Ea_sym4

**Table 4.** Area under the ROC curve (AUC), CA, F1, precision and recall from all three ML methods (kNN, Random Forest and SVM) performed in the two forms of data analysis (five rank and all features) on T1-weighted, T1-weighted with contrast, T2-weighted, DWI and FLAIR images.

Sequence	Feature	Method	AUC	CA	F1	Precision	Recall
*T1*	Five rank	kNN	0.838	0.806	0.857	0.814	0.906
		Random Forest	0.906	0.827	0.875	0.837	0.912
		SVM	0.850	0.750	0.835	0.724	0.987
	All features	kNN	0.815	0.802	0.797	0.799	0.802
		Random Forest	0.835	0.790	0.784	0.786	0.790
		SVM	0.732	0.737	0.693	0.781	0.737
*T1 C+*	Five rank	kNN	0.722	0.745	0.816	0.742	0.891
		Random Forest	0.761	0.779	0.857	0.806	0.914
		SVM	0.691	0.766	0.85	0.794	0.914
	All features	kNN	0.682	0.735	0.8105	0.738	0.883
		Random Forest	0.708	0.745	0.835	0.784	0.892
		SVM	0.667	0.757	0.793	0.792	0.801
*T2*	Five rank	kNN	0.636	0.716	0.618	0.697	0.716
		Random Forest	0.794	0.783	0.776	0.775	0.783
		SVM	0.553	0.615	0.618	0.621	0.615
	All features	kNN	0.621	0.963	0.680	0.674	0.693
		Random Forest	0.774	0.757	0.748	0.746	0.757
		SVM	0.530	0.674	0.586	0.572	0.674
*DWI*	Five rank	kNN	0.705	0.679	0.676	0.677	0.679
		Random Forest	0.752	0.683	0.681	0.681	0.683
		SVM	0.717	0.718	0.704	0.738	0.718
	All features	kNN	0.705	0.679	0.676	0.677	0.679
		Random Forest	0.706	0.675	0.670	0.674	0.675
		SVM	0.689	0.698	0.688	0.705	0.698
*FLAIR*	Five rank	kNN	0.763	0.744	0.735	0.735	0.744
		Random Forest	0.757	0.719	0.713	0.710	0.719
		SVM	0.640	0.610	0.618	0.656	0.606
	All features	kNN	0.693	0.670	0.660	0.658	0.670
		Random Forest	0.753	0.714	0.699	0.704	0.714
		SVM	0.625	0.606	0.614	0.650	0.606

## Discussion

In the view of fast innovation and development of diagnostic equipment, the volume and complexity of diagnostic images increase every day. Thus, researchers worldwide frequently are searching for algorithms to facilitate the diagnosis and to minimize the costs for the institution [23-27]. In this context, our tool aims to assist the radiologists, bringing greater safety to the diagnosis, agility, and reducing costs.

**Figure 3.**
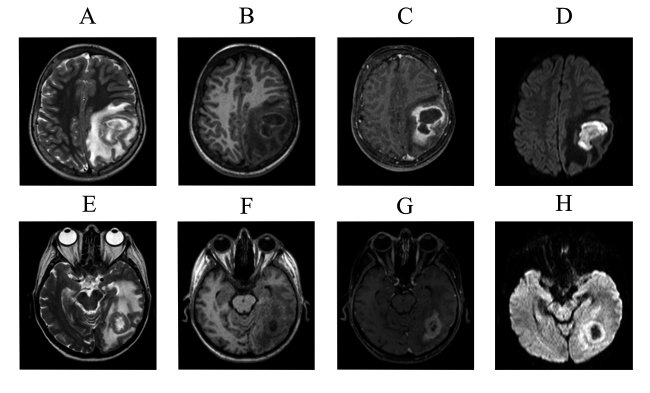
In upper images, a patient with pyogenic abscess, displayed with different MRI sequences. **(A)** T2-weighted images. **(B)** T1-weighted images without contrast. **(C)** T1-weighted images with gadolinium contrast medium. **(D)** DWI diffusion weighted images. In bottom images, a patient with a primary brain tumor, with the same MRI sequences. **(E)** T2-weighted images. **(F)** T1-weighted image with contrast. **(G)** T1-weighted image with gadolinium contrast medium. **(H)** DWI diffusion weighted image. Both have a pattern of a cerebral ring enhancing lesion. In abscess there is restricted diffusion in the core **(D)** a feature not demonstrated in the tumoral lesion.

The difficulty to differentiate between inflammatory and tumoral lesions was demonstrated by many papers [28-31]. Figure 3 illustrates two patients, one diagnosed with pyogenic abscess (upper images A - D) and the other with a primary brain tumor (bottom images E - H). The pyogenic abscess appears as a large cortico-subcortical parietal rounded lesion, and the brain tumor as an occipital cortico-subcortical lesion. Both lesions present similar signal intensities: hyperintense core with hypointense halo on T2 weighted-images, hypointense on T1 weighted-images with peripheral enhancement. Both are surrounded by extensive edema and mass effect. Usually, inflammatory and tumoral lesions are hyperintense on T2 and hypointense on T1 (as the most part of brain lesions) and may demonstrate peripheral ring enhancement. In this case, restricted diffusion in the core of the lesion yielded the correct diagnosis of pyogenic abscess. However, the absence of restricted diffusion in the core may occur in tumors or inflammatory lesions.

Perfusion sequences in MRI can be used in this differential diagnosis since they provide important physiologic and pathophysiologic parameters and can be assessed non-invasively. There are several techniques to obtain perfusion-related parameters using endogenous contrast methods or, more robustly and more widely used, exogenous gadolinium-based contrast agent dynamic methods [7]. However, many patients have a contraindication to gadolinium, and inflammatory lesions, such as neurotuberculosis, may show high perfusion, leading to misdiagnosis. Thus, the potential use of other tools must be investigated to differentiate neuroinfections from tumoral lesions. 

In this context, our approach combined statistical textural features with methods of ML and was able to differentiate neuroinfections from brain tumors in MRI sequences. We compared the classification between five MRI sequences: T1-weighted images, T1-weighted with contrast medium, T2-weighted images, DWI, and FLAIR. We also compared the results changing the input data (all features and five best-ranked) to achieve the best classification.

Our results showed differences in the classification efficiency between T1, T2, DWI, and FLAIR in MRI images. These differences are most likely due to differences in the parameters used in the acquisition and processing steps. Studies show that parameters used for imaging acquisition, including repetition time, echo-time [32], and spatial resolution [33], can influence the extraction and the quality of textures.

The ROC curve for the SVM presented a random guessing line behavior, which means that the classification was not that efficient. We also observed that SVM had high recall values (0.987 for T1 and 0.914 for T1C+). These results reflect a high rate of true positives. However, the majority of F1-score indicated low specificity, which show a high rate of false positives. In our classification system, high true positives and false positives mean that SVM classified a high number of patients with brain tumors correctly, but also misclassified a large number of patients with inflammatory lesions as brain tumors. T1-weighted images proved to be the best sequence for classification. The Random Forest classifier presented a reliable behavior with those T1 images. That reflected on high accuracy (0.827), Area under ROC curve (0.906), precision (0.837) and recall (0.912).

According to Carter et al, 2016, the ROC curve AUC is a function of sensitivity and specificity of the prediction model that can rank the test as excellent (AUC higher than 0,9), strong (AUC higher than 0.8), reasonable (AUC higher than 0.7) or non-useful (AUC below 0.7 [22]. Based on this, we can classify the approach with all the features in Random Forest as strong (T1) and reasonable (T1 with contrast medium, T2, DWI, and FLAIR). The approach with only the five best-ranked features was classified as excellent (T1), and reasonable (T1 with contrast medium, T2, DWI, and FLAIR) in Random Forest classification. Thus, our classification model using the best-ranked features extracted from T1-weighted MRI images showed great classification potential. Sequences T2-weighted, FLAIR, DWI, and T1 with contrast medium did not achieve the same classification performance.

In all MRI sequences, the best-ranked features show at least reasonable AUC, CA, F1, precision, and recall in all three methods (SVM, kNN, and Random Forest). The five best-ranked features were different in each MRI image. For T1-weighted images, the five best were EntropyWv, Mean, Ed_haar_2, Ev_haar_2 and Ev_sym4_2. The five best-ranked features are considered best for the classification model in opposite to using them all.

Since our goal was to find the best set of training data to improve classification, the information in excess can cause noise. This noise leads the model to learn random patterns that do not improve its ability to sort images correctly. This excess of information is called overfitting and is a well know problem in ML [34]. Therefore, the best-ranked features can be used as a strategy to avoid overfitting and improve and optimize the model.

Image processing and texture analysis have been progressively used to differentiate subtypes of tumors [12, 14, 16, 35] and infections [14-19], this shows us that the subtypes are heterogeneous. Our research presented a model for the differentiation of two different generic classes of pathologies, which represented several subtypes of diseases (as showed in Table 1). The fact that we were able to correctly separate classes “inflammatory lesions” and “brain tumors” points to the possibility that, although heterogeneous in the subtypes, these pathologies have characteristics similar enough to be correctly grouped based only on features extracted from the images.

The correct diagnosis between tumors and inflammatory lesions, especially in the CNS, can influence in the surgery, course of treatment, and prognosis of the patient. The biopsy is a very important diagnostic tool. However, a biopsy can lead to the spread of the infection in the parenchymal tissue. Some tumors present high seeding risk and the biopsy can increase the metastasis risk [36]. The correct differentiation using noninvasive methods can lower the risk of complications, the spread of the disease, and morbidity. This work may represent another important tool (such as spectroscopy or perfusion) to be used in the radiological practice, as it showed the potential to distinguish among these groups. Still, due to the relatively small number of cases, this work initiates the discussion about the use of this method and potential further research with broader samples.

## Conclusion

In the present study, we developed an approach based on the association of texture analysis and machine learning classifiers that differentiated inflammatory lesions from tumors. It is believed that the five best-ranked features were more efficient than all the features combined for classification. In our model, we achieved the best results with the Random Forest classifier on T1-weighted images. The classification combined with the radiologist experience and other patient data (family history, symptoms, lifestyle, and others) may aid and improve the diagnosis of lesions with a similar pattern of a cerebral ring enhancing on MRI.

### Abbreviations

AUC: area under the curve; CA: accuracy; CNS: central nervous system; CSF: cerebrospinal fluid; CT: Computed Tomography; DWI: diffusion weighted image; F1: F-score; FLAIR: fluid attenuated inversion recovery; GLCM: gray-level co-occurrence matrix; GLRL: gray-level run length; kNN: k-nearest neighbors; ML: machine learning; MRI: magnetic resonance imaging; PET: positron emission tomography; ROC: receiver operating characteristic; ROI: region of interest; SVM: support vector machine; T1 C+: T1-weighted sequence with contrast medium; TA: texture analysis.

## Supplementary material

The following online material is available for this article:

Additional file 1. Complete list of patients with inflammatory lesions including their ages, gender, and diagnostic evaluation.

Additional file 2. Complete list of patients with tumors including their ages, gender, and diagnostic evaluation.
