# Supplementary material for: Inflammatory lesions and brain tumors: is it possible to differentiate them based on texture features in magnetic resonance imaging?
Source: J Venom Anim Toxins Incl Trop Dis. 2020 Sep 4;26:e20200011. doi: 10.1590/1678-9199-JVATITD-2020-0011 (PMC7473508; doi:10.1590/1678-9199-JVATITD-2020-0011)
Supplement: Additional file 1. [file 1678-9199-jvatitd-26-e20200011-s1.pdf]

J Venom Anim Toxins incl Trop Dis
https://doi.org/10.1590/1678-9199-JVATITD-2020-0011

Supplementary Material to "Inflammatory lesions and brain tumors: is it possible to differentiate them based on texture features in magnetic resonance imaging?"

Additional file 1. Complete list of patients with inflammatory lesions including their ages, gender, and diagnostic evaluation.

Patients	Age	Gender	Edema	Diagnosis	Confirmation
**1**	40	M	Marked	Toxoplasmosis	Biopsy
**2**	59	F	Marked	Cryptococcosis	Biopsy
**3**	20	F	Marked	Moderate neuromyelitis	CFS
**4**	56	F	Marked	Neurocysticercosis	*
**5**	45	F	Marked	Pyogenic abscess	Biopsy and culture
**6**	43	F	Marked	Aspergillosis	Biopsy
**7**	62	F	Marked	Aspergillosis	Biopsy
**8**	15	F	Mild	Multiple sclerosis	Dissemination in time and space + CSF analysis
**9**	41	F	Mild	Septic emboli	Clinical and radiological correlation and radiological evolution
**10**	37	F	Marked	Tuberculosis	Biopsy
**11**	37	M	Marked	Toxoplasmosis	Biopsy
**12**	5	M	Marked	Pyogenic abscess	Biopsy and culture
**13**	41	F	Marked	Toxopasmosis	Biopsy
**14**	32	F	Marked	Neurocysticercosis	*
**15**	32	F	Marked	Toxoplasmosis	Biopsy
**16**	45	F	Mild	Neurocysticercosis	*
**17**	56	M	Marked	Progressive multifocal leukoencephalopathy	Biopsy

Patients	Age	Gender	Edema	Diagnosis	Confirmation
**18**	25	M	Mild	Septic emboli	Clinical and radiological correlation and radiological evolution
**19**	24	F	Mild	Multiple sclerosis	Dissemination in time and space + CSF analysis
**20**	52	M	Mild	Toxoplasmosis	Biopsy
**21**	41	F	Mild	Toxoplasmosis	Biopsy
**22**	43	M	Mild	Pyogenic abscess	Biopsy
**23**	18	F	Mild	Multiple sclerosis	Dissemination in time and space + CSF analysis
**24**	29	F	Mild	Cryptococcosis	CSF
**25**	44	F	Mild	Toxoplasmosis	Biopsy
**26**	42	M	Mild	Toxoplasmosis	Biopsy
**27**	33	M	Mild	Tuberculosis	CSF
**28**	36	F	Mild	Tuberculosis	CSF
**29**	65	M	Marked	Tuberculosis	Biopsy
**30**	53	F	Marked	Vasculitis	Biopsy

*Single or multiple active parenchymal cysts, with at least one cyst with scolex on MRI.
