# Supplementary material for: Inflammatory lesions and brain tumors: is it possible to differentiate them based on texture features in magnetic resonance imaging?
Source: J Venom Anim Toxins Incl Trop Dis. 2020 Sep 4;26:e20200011. doi: 10.1590/1678-9199-JVATITD-2020-0011 (PMC7473508; doi:10.1590/1678-9199-JVATITD-2020-0011)
Supplement: Additional file 2. [file 1678-9199-jvatitd-26-e20200011-s2.pdf]

J Venom Anim Toxins incl Trop Dis
https://doi.org/10.1590/1678-9199-JVATITD-2020-0011

Supplementary Material to "Inflammatory lesions and brain tumors: is it possible to differentiate them based on texture features in magnetic resonance imaging?"

Additional file 2. Complete list of patients with tumors including their ages, gender, and diagnostic evaluation.

Patient	Age	Gender	Edema	Diagnosis
**1**	48	F	Marked	Glioblastoma
**2**	40	F	Mild	Glioblastoma
**3**	76	F	Marked	Glioblastoma
**4**	45	M	Mild	Glioblastoma
**5**	31	M	Mild	Glioblastoma
**6**	68	M	Mild	Grade III astrocytoma
**7**	22	F	Mild	Grade II astrocytoma
**8**	37	M	Mild	Ependymoma
**9**	54	M	Marked	Glioblastoma
**10**	55	M	Marked	Glioblastoma
**11**	50	M	Mild	Gliosarcoma
**12**	53	F	Marked	Glioblastoma
**13**	17	F	Mild	Grade II astrocytoma
**14**	67	M	Mild	Grade II astrocytoma
**15**	74	F	Marked	Glioblastoma
**16**	35	F	Mild	Grade III astrocytoma
**17**	52	F	Mild	Glioblastoma
**18**	45	M	Mild	Glioblastoma
**19**	67	M	Mild	Glioblastoma
**20**	76	M	Mild	Grade III astrocytoma
**21**	52	F	Marked	Grade III astrocytoma
**22**	67	F	Marked	Gliosarcoma
**23**	42	F	Mild	Grade III astrocytoma
**24**	58	M	Mild	Grade II astrocytoma
**25**	34	F	Mild	Grade II astrocytoma
**26**	35	F	Mild	Grade II astrocytoma
**27**	32	M	Mild	Grade II astrocytoma
**28**	23	F	Mild	Grade II astrocytoma
**29**	58	M	Mild	Grade III astrocytoma
**30**	45	M	Mild	Grade III astrocytoma
**31**	31	F	Mild	Grade III astrocytoma
**32**	31	M	Mild	Grade III astrocytoma

Patient	Age	Gender	Edema	Diagnosis
**33**	39	M	Mild	Grade III astrocytoma
**34**	77	M	Marked	Grade III astrocytoma
**35**	56	F	Mild	Glioblastoma
**36**	28	m	Marked	Glioblastoma
**37**	21	F	Marked	Glioblastoma
